# Per- and Polyfluoroalkyl Substances (PFAS) Within the Exposome: Cellular and Molecular Mechanisms Underlying a Potential Risk for Cardiac Arrhythmias and Atrial Fibrillation?

**DOI:** 10.3390/cells15080696

**Published:** 2026-04-15

**Authors:** Mikaelys Plantier, Nour Naji, Andréane Dupont, Roddy Hiram

**Affiliations:** 1 Montreal Heart Institute, Montreal, QC H1T 1C8, Canada; 2 Faculty of Exact and Natural Sciences, Université des Antilles, 97110 Pointe-à-Pitre, Guadeloupe, France; 3 Department of Medicine, Faculty of Medicine, Université de Montréal, Montreal, QC H3T 1J4, Canada; 4 Department of Chemistry, Université du Québec à Montréal, Montreal, QC H2L 2C4, Canada

**Keywords:** PFAS, pollutants, cardiovascular disorders, atrial fibrillation, inflammation

## Abstract

**Highlights:**

**What are the main findings?**

- PFAS were shown to promote myocardial inflammation and fibrosis.
- Preclinical reports pointed a deleterious role of PFAS exposure on Ca^2+^ currents.

**What is the implication of the main finding?**

- Prolonged exposure to PFAS is responsible for ECG remodeling and arrhythmogenesis.
- Preclinical and basic research are required to elucidate the mechanisms of PFAS toxicity on arrhythmias’ onset.

**Abstract:**

**Background**: Per- and polyfluoroalkyl substances (PFAS) represent a large class of synthetic fluorinated compounds characterized by highly stable carbon–fluorine bonds that confer exceptional environmental persistence and bioaccumulative properties. Although regulatory measures have restricted the production of several PFAS, including perfluorooctanoic acid (PFOA) and perfluorooctanesulfonic acid (PFOS), their environmental persistence continues to maintain widespread human exposure, while newly introduced replacement compounds raise additional toxicological concerns. Notably, the recent evidence demonstrating PFAS-induced alterations in key cardiac ion channel activity and electrocardiographic parameters suggest potential electrophysiological mechanisms that may contribute to arrhythmogenesis and cardiac arrhythmias including the most frequent one, atrial fibrillation (AF). **Methods**: We conducted a narrative literature review of experimental, epidemiological, and mechanistic studies investigating and reporting the cardiovascular, electrophysiological, and potential arrhythmogenic effects of PFAS. **Results**: Available evidence indicates that PFAS exposure is associated with alterations in cardiac electrophysiology, including modulation of ion channel activity (notably sodium, calcium, and potassium channels), disruption of calcium handling, and changes in electrocardiographic parameters such as QT interval prolongation, which are key contributors to arrhythmogenesis and AF. **Conclusions**: This review highlights the need for improved understanding of PFAS-induced electrophysiological alterations, to clarify the role of PFAS in cardiac arrhythmias including AF.

## 1. Introduction

Per- and polyfluoroalkyl substances (PFAS) constitute a large family of synthetic fluorinated compounds characterized by exceptional chemical stability and resistance to degradation [1]. The high persistence of PFAS has led to widespread industrial and consumer use in various applications such as firefighting foams, non-stick cookware (Teflon), food packaging, cosmetics, and stain-resistant fabrics [2]. Because of their highly recalcitrant property, PFAS are often called “Forever Chemicals” [3]. The chemical structure of PFAS includes strong carbon–fluorine bonds (C–F) conferring extreme stability, high hydrophobia, and exceptional oleophobic property responsible for environmental persistence and alarming bioaccumulation in various organisms including humans [1].

Routes for human exposure to PFAS include the air (industrial emissions), soils (agriculture), water (bath and drinking), dermal contact (cosmetics, medical devices), and ingestion (food or water consumption, pharmaceutical medications, medical implants) [4]. The widespread detection of PFAS in human serum and breast milk globally underscores growing concerns about their long-term health consequences [5]. Studies converge to designate PFAS as emerging contributors of various severe disorders including—among others—infertility, developmental issues, as well as prostate, kidney, thyroid, breast, and ovarian cancers.

PFAS-related cardiovascular toxicity is increasingly recognized, with accumulating evidence associating exposure with lipid metabolism disorders such as dyslipidemia, hypertension, impairment of endothelial function, and a higher risk of developing cardiovascular diseases (CVD) [6]. Following exposure, PFAS bind extensively to serum proteins, primarily albumin and globulin, facilitating their prolonged circulation in the bloodstream and systemic distribution to highly perfused, protein-rich organs, including the heart, thereby promoting a mechanistic link between environmental exposure and potential cardiac effects [7,8]. However, few data are available about the potential effects of PFAS on cardiac electrical activity and arrhythmogenesis. Cardiac arrhythmias such as atrial fibrillation (AF) arise from a combination of electrical, structural, and inflammatory mechanisms, which nourish the arrhythmogenic substrate [9]. Then, it is plausible that PFAS-induced physiological disorders and cardiotoxic pathways may intersect with known arrhythmogenic substrates.

This narrative review critically examines current evidence linking PFAS exposure to cardiac arrhythmias, integrating epidemiological findings, experimental models, and mechanistic insights.

The main objectives of this narrative reviews are to discuss: (i) the epidemiological evidence of PFAS’ implication in the development of arrhythmogenic cardiac disorders, (ii) the experimental evidence of PFAS-induced arrhythmogenic substrate, (iii) the potential underlying mechanisms linking PFAS exposure and the development of cardiac arrhythmias, and (iv) the comparison of PFAS-related toxicity pathways with those of selected other environmental contaminants.

Overall, we discuss and highlight the current knowledge gaps relevant to environmental cardiology and the human exposome in the specific context of PFAS exposure and cardiac arrhythmias including AF.

## 2. Methods

A narrative literature review was conducted to identify relevant clinical and experimental studies examining the relationship between per- and polyfluoroalkyl substances (PFAS), cardiovascular disease, and cardiac arrhythmias, with a particular focus on atrial fibrillation (AF). Electronic searches were performed in PubMed, MEDLINE, Scopus, and ScienceDirect from database inception until April 2026.

The search strategy was structured using Boolean operators and appropriate syntax as follows: (“PFAS” OR “perfluoroalkyl substances” OR “polyfluoroalkyl substances”) AND (“cardiac” OR “heart” OR “cardiovascular”) AND (“atrial fibrillation” OR “cardiac arrhythmias”) AND (“inflammation” OR “fibrosis” OR “electrophysiology” OR “ion channels”).

Eligible studies included original research articles (clinical, epidemiological, in vivo, and in vitro experimental studies) as well as relevant review articles providing mechanistic or translational insights. Only articles published in English with full-text availability were considered. Studies were included if they addressed PFAS exposure and cardiovascular outcomes, with particular emphasis on cardiac electrophysiology, arrhythmias, or related mechanistic pathways. Articles not directly related to cardiovascular or electrophysiological outcomes could be considered for discussion purposed, but non-peer-reviewed reports, and studies lacking sufficient methodological detail were excluded.

Titles and abstracts were initially screened for relevance, followed by full-text review of selected articles. Additional studies were identified through manual screening of reference lists of included articles. The final selection of studies was based on relevance to the objectives of the review and their contribution to understanding the mechanistic and clinical links between PFAS exposure and cardiac arrhythmias.

## 3. Epidemiological Evidence of PFAS’ Implication in the Development of Arrhythmogenic Cardiac Disorders

### 3.1. Generalities

PFAS are a chemical family of thousands of organic compounds [1]. Among the most measured in the population, we found perfluorooctanoic acid (PFOA), perfluorononanoic acid (PFNA), perfluorodecanoic acid (PFDA), perfluorohexane sulfonate (PFHxS), perfluorooctane sulfonate (PFOS), or trifluoroacetic acid (TFA) [10]. Mounting evidence suggests that PFAS are responsible for myocardial remodeling and CVD events, though the studies reporting a link with cardiac arrhythmias are sparse [11]. This section aims to review current clinical evidence of the implication of PFAS in the development of major recognized cardiac risk factors for ventricular and atrial rhythm disorders including AF.

### 3.2. PFAS Exposure Is Associated with Major Risk Factors for Arrhythmias Including AF

In 1229 participants in which the serum levels of 13 PFAS exceeded 85% of detection rate, the analysis of electrocardiogram (ECG) parameters revealed a significant reduction in QRS duration and augmentation of PR interval [12]. In a retrospective study involving 46,553 patients exposed to high levels of PFOS and PFHxS in drinkable water, were significantly more vulnerable to acute myocardial infarction (MI) and hemorrhagic stroke in patients aged 50–75 years old, while the risk of CVD morbidity and mortality increased in patients above 75 years old [13]. In a prospective study involving 710 patients, elevated levels of PFOS and PFOA were associated with an increased risk of acute coronary syndrome (ACS) [14]. A retrospective study involving 101 patients revealed that increased plasmatic levels of PFOS were associated with a higher risk of coronary heart disease [15]. A multicenter study involving 432 patients has shown that prenatal exposure to 13 PFAS present in maternal plasma revealed a significant association with the development of congenital heart disease (CHD) [16]. The retrospective study of 7904 patients revealed that elevated serum levels of PFAS, including PFOS, are accompanied by increased risk of CVD and stroke [17]. In a cross-sectional study involving 801 patients aged 70 years old and above, increased plasmatic levels of PFAS, especially PFNA, were associated with cardiac remodeling characterized by a significant reduction in the myocardial wall thickness and significantly elevated left ventricular diameter [18]. In terms of differences related to biological sex, a study involving 42,742 patients revealed that exposure to PFAS, including PFOS and PFOA, was accompanied by increased risk of ischemic heart disease where women were more susceptible to PFOA and PFOS toxicity, but the survival rate was significantly worse in men [19]. In contrast, in a retrospective study involving 1528 patients in which blood samples were collected in 2003–2009 and 1997–1999 with follow-up through 2017 and 2014, the levels of 5 quantifiable PFAS were not associated with the increased risk of MI and stroke [20] (Table 1).

It is well-accepted that arrhythmias and AF are multifactorial and driven by a combination of established clinical and biological risk factors [21]. Underlying cardiovascular conditions such as CVD, heart failure, coronary artery disease, CHD, ACS or valvular heart disease are recognized contributors increasing the risk of arrhythmias and AF susceptibility [22].

Longitudinal evidence directly linking PFAS exposure to AF is currently sparse. It is, however, biologically plausible that the cardiac disorders caused by PFAS might lead to a higher risk of arrhythmias’ incidence. While several prospective studies have demonstrated associations between PFAS and established risk factors for arrhythmogenesis, investigations specifically addressing incident AF remain limited. This gap highlights the need for longitudinal studies incorporating precise exposure assessment and robust arrhythmia phenotyping.

## 4. Experimental Evidence of PFAS-Induced Arrhythmogenic Substrate

### 4.1. Prerequisites

Data from basic research performed in vitro on cardiac cells, or in vivo in fish, mice, or rats, provide a translational platform to assess cardiotoxic effects of environmental chemicals. PFAS compounds have been shown to alter heart rate and repolarization dynamics [23,24,25]. Targeted mechanistic studies indicate that certain PFAS interfere with key cardiac ion channels’ calcium currents [26,27,28,29]. In addition, PFAS was suggested to provoke myocardial hypertrophy, inflammation and fibrosis [29]. These functional changes align with recognized pro-arrhythmic phenotypes [21]. This section aims to review recent results from basic research that may support the development of arrhythmogenic substrate following PFAS exposure.

### 4.2. PFAS Exposure Provokes Cardiac Remodeling Potentially Involved in the Arrhythmogenic Substrate

A study involving human induced pluripotent stem cell-derived cardiomyocytes (hiPSC-CMs) revealed that 14 days exposure to a mixture combining PFDA, PFOA, and PFHxS (20–200 μM) provoked important cardiomyocyte (CM) remodeling, including altered mitochondrial membrane potential and function, increased oxidative stress, reduced ATP content, increased secretion of fibrosis biomarkers, and reduced α-actinin [23]. PFOS, PFOA, PFNA and PFHxS were shown to have embryotoxic effects by provoking significantly abnormal expression of genes involved in CM differentiation and myocardial development and function [24]. A study of 56 PFAS revealed that in vitro exposure to the ‘Forever Chemical’ provoked QT prolongation and cardiotoxicity in hiPSC-CMs [25].

In isolated guinea-pig ventricular myocytes, whole-cell patch-clamp recording revealed that 10 µM PFOS and PFOA could decrease cellular action potential duration, increase voltage-activated peak amplitude of L-type calcium (Ca^2+^) currents and shift the half-activation and inactivation voltages of L-type Ca^2+^ currents to hyperpolarization [26]. Such results converge with other reports suggesting that PFOS induces CM toxicity by provoking mitochondrial structure damages and abnormal Ca^2+^ shuttle [27]. The QT prolongation reported in the hiPSC-CMs study should be interpreted as an increase in action potential duration (APD) [25], a cellular surrogate of ventricular repolarization, for which consistent confirmation at the whole-organism level following PFAS exposure remains limited, and which can be mechanistically reconciled with the reported reduction in L-type Ca^2+^ current by considering the net balance of ionic currents [27], where concomitant inhibition of repolarizing potassium currents may ultimately delay repolarization.

In zebrafish embryos, PFOS exposure decreased the heart rate [28]. In *Oryzias melastigma* embryos, PFOS exposure was shown responsible for cardiac malfunction, altered heart rate, and increased expression of inflammation and fibrosis markers cyclooxygenase-2 (COX-2) and fibroblast growth factor 8 (FGF8) [29]. A study involving adult male Sprague Dawley rats exposed to 10 mg/kg PFOS injected intraperitoneally daily for 14 days, revealed increased myocardial damage as identified by elevated myocardial levels of cardiac troponin-T, and serum levels of lactic dehydrogenase, creatine kinase, and creatine kinase-isoenzyme-MB [30]. In this study, the authors also observed increased myocardial hypertrophy, cardiac fibrosis, and augmentation of inflammation biomarkers (IL1β, TNFα) in the rat heart tissues [30]. Mice and rats exposed to 10 and 20 mg/kg PFOS were affected by a significant enlargement of the right atrium (RA) [31] (Table 2).

The direct link between PFAS exposure and cardiac arrhythmias, including AF, remains insufficiently explored. Available evidence suggests that PFAS may contribute to impaired Ca^2+^ handling, altered action potential dynamics, abnormal heart rate, myocardial deformation, as well as cardiac inflammation and fibrosis [26,29]. Such alterations are well known to promote the development and maintenance of an arrhythmogenic substrate in the myocardium [21]. Collectively, these findings warrant further investigation to better define the potential role of PFAS in the pathogenesis of cardiac arrhythmias.

### 4.3. Translational Gap in PFAS Research: Importance of Dose-Relevant Mechanistic Studies

While some hiPSC-CMs studies and in vivo models such as zebrafish report electrophysiological alterations at both nanomolar and micromolar concentrations, rodent studies often demonstrate cardiac effects primarily at higher, micromolar levels (Table 2). In contrast, typical human exposures are generally within the low nanomolar range, although substantially higher levels may occur in heavily contaminated settings. Together, these discrepancies highlight a critical dose-dependent gap in the current literature and underscore the need for further mechanistic studies to better define the relevance of PFAS exposure to cardiac arrhythmogenesis (Table 2).

Biomonitoring studies consistently show that PFAS are detectable in the blood of the vast majority of the general population, with circulating concentrations typically in the low nanomolar range [17,18,19,20]. PFOS and PFOA are among the most prevalent compounds, with average serum levels generally around ~10 ng/mL [17,18].

In highly exposed populations, such as those affected by contaminated drinking water or occupational exposure, serum concentrations can rise from tens to hundreds of ng/mL, and in rare cases may approach the upper nanomolar to low micromolar range [32]. However, such levels remain exceptional and are typically observed under conditions of severe environmental contamination. It is, therefore, important to study whether such episodes of severe contamination correlate with increased CVD event, including cardiac arrhythmias such as AF.

Overall, these data indicate that typical human PFAS exposure occurs at low nanomolar concentrations, substantially lower than the micromolar ranges frequently used in experimental studies [17,18,19,20]. This discrepancy is important when interpreting the physiological relevance of reported cellular effects, while also recognizing that extreme exposure scenarios may partially overlap with concentrations used in some in vitro and in vivo models.

## 5. Emerging Mechanistic Insights

### 5.1. Preamble

Emerging preclinical and clinical evidence suggests that PFAS may contribute to myocardial remodeling and cardiac disease by inducing deleterious electrical, structural, and functional alterations consistent with the typical characteristics of an arrhythmogenic substrate. This section aims to provide a rationale for the hypothesis that PFAS may promote cardiac arrhythmias, including AF, which remain insufficiently explored in this context.

### 5.2. PFAS-Induced Electrophysiological Remodeling

**Figure 1:**
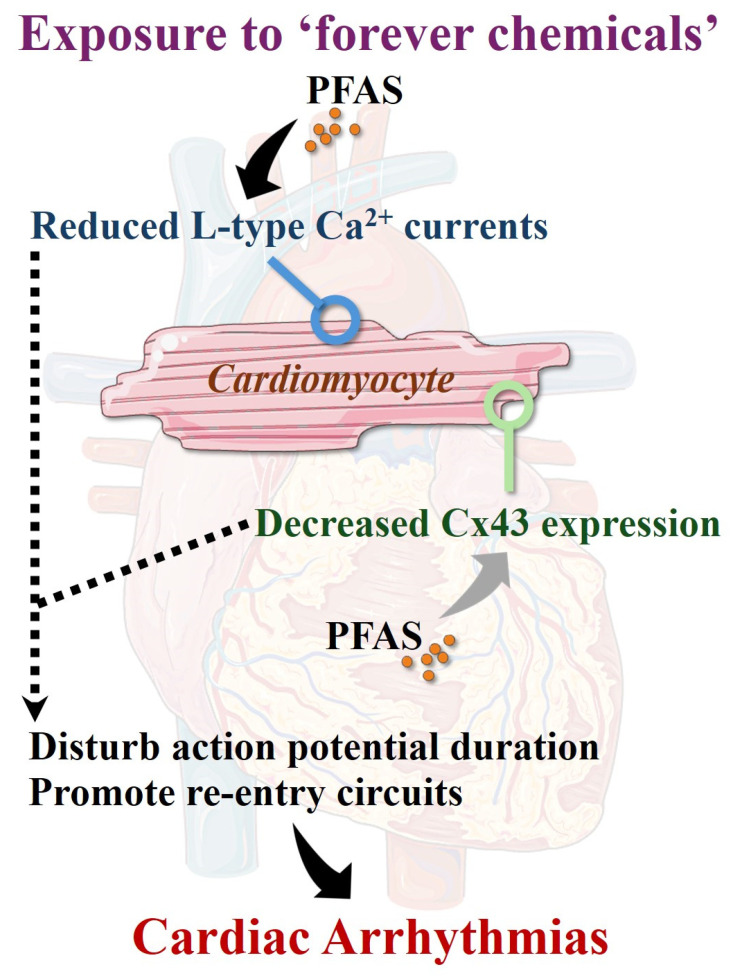
PFAS exposure provokes electrical remodeling affecting cardiomyocytes. Mounting evidence suggests that prolonged exposure to PFAS might alter L-type Ca^2+^ currents in CM. In addition, PFAS were shown to reduce Cx43 expression. Such events are described as arrhythmogenic substrates of cardiac arrhythmias. Dark arrows: demonstrated link. Gray arrow: suggested effect in CM based on demonstrated impact in non-cardiac cells. Dashed arrows: demonstrated effect on cardiac arrhythmias. **Abbreviation**: Ca^2+^: calcium; CM: cardiomyocyte: Cx43: connexin 43; PFAS: per- and polyfluoroalkyl substances.

Disruption of key myocardial ion currents can alter action potential duration, conduction velocity, and refractoriness, thereby facilitating triggered activity or re-entry [33]. The above-described role of PFAS in provoking alterations of L-type Ca^2+^ currents [26] suggests a potential mechanistic link with arrhythmogenesis at the cellular level [34]. It has been shown that downregulation of L-type Ca^2+^ current is an important determinant of electrical remodeling in cardiac arrhythmias including chronic AF [34]. Studies have shown that CM displaying reduced L-type Ca^2+^ current density are more vulnerable to AF [35]. Reduced L-type Ca^2+^ current diminishes Ca^2+^ influx, leading to shortened action potential duration and reduced effective refractory period, which facilitates re-entry [34]. Concurrently, disrupted intracellular Ca^2+^ handling, characterized by sarcoplasmic reticulum calcium leak, impaired reuptake via sarcoendoplasmic reticulum calcium ATPase (SERCA), promoting delayed afterdepolarizations and triggered activity [34,35] (Figure 1).

Connexin-43 (Cx43) is a key gap junction protein that enables CM-CM electrical coupling and supports rapid impulse propagation [36]. In non-cardiac tissue, PFOA and PFOS were shown to significantly reduce Cx43 expression [37]. Alterations in Cx43 expression and distribution, including lateralization from intercalated disks, impair cell-to-cell conduction and promote conduction slowing and heterogeneity [38]. Hence, abnormal Cx43 expression and activity favor wavefront fragmentation and the formation of re-entry circuits, thereby increasing susceptibility to cardiac arrhythmias including AF [39].

Together, these PFAS-induced Cx43 electrical changes and Ca^2+^-handling abnormalities might contribute to both the initiation and maintenance of cardiac tachyarrhythmias including AF (Figure 1).

### 5.3. PFAS-Induced Inflammation and Oxidative Stress

Experimental evidence suggests that PFAS can increase the production of reactive oxygen species and activate pro-inflammatory signaling pathways, leading to cytokine release and cellular injury [23,29]. These processes can promote structural remodeling, including fibrosis, a driver of arrhythmogenesis and AF [39]. Patients exposed to chronic PFAS ingestion through drinkable water were more vulnerable to MI [13].

**Figure 2:**
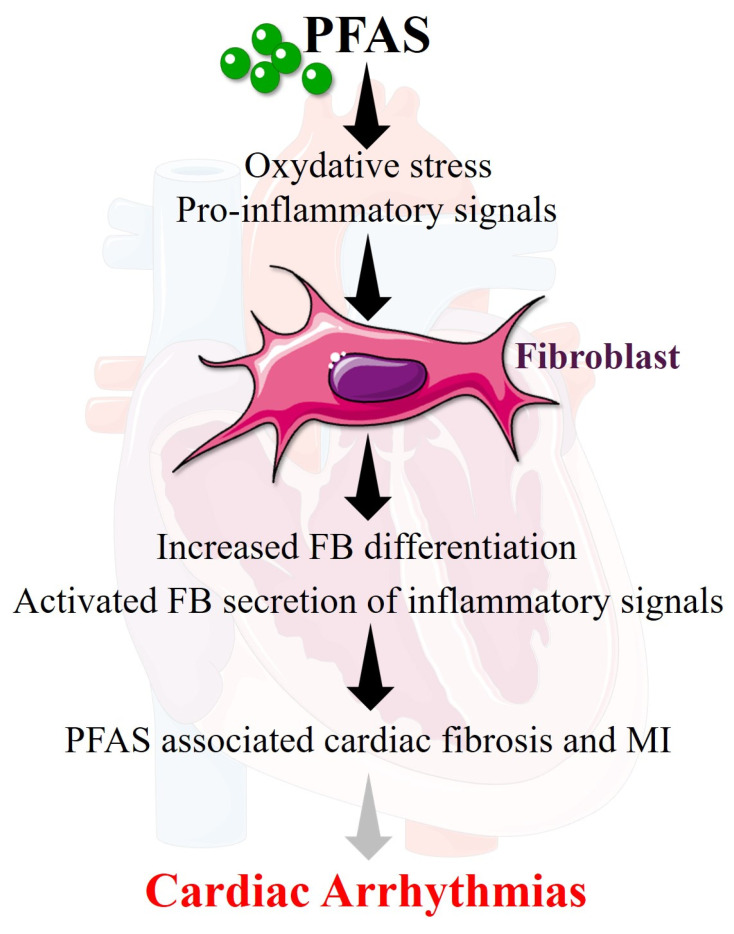
PFAS might promote arrhythmogenic fibroblast inflammation and differentiation. Converging evidence suggests that PFAS exposure is responsible increase expression of reactive oxygen species promoting oxidative stress and activation of inflammation. Such events are recognized promotors of FB-differentiation and FB-triggered fibrosis, which might explain why patient exposed to PFAS were found significantly more at risk of MI, a major risk factor for cardiac arrhythmias. **Dark arrows**: demonstrated link. **Gray arrow**: suggested effect based on demonstrated impact of PFAS in the development of recognized arrhythmogenic remodeling. **Abbreviation**: FB: fibroblast; MI: myocardial infarction; PFAS: per- and polyfluoroalkyl substances.

Inflammatory processes and signals (e.g., NLRP3 inflammasome, IL1β, TNFα, COX2) contribute to arrhythmogenesis by driving fibrotic remodeling and electrical dysfunction [39]. Cytokine-mediated activation of fibroblasts enhances extracellular matrix deposition, leading to myocardial fibrosis that disrupts electrical conduction continuity [40]. Such myocardial structural remodeling, together with inflammation-induced alterations in ion channels and connexins, increases conduction heterogeneity and promotes re-entrant circuits, key mechanisms underlying arrhythmias including AF [39,41] (Figure 2).

Collectively, PFAS-induced inflammatory and oxidative responses may contribute to the development of a pro-arrhythmic substrate and increase susceptibility to cardiac rhythm disorders including AF.

### 5.4. PFAS-Induced Structural Remodeling

Exposure to PFAS has been increasingly linked to cardiac structural remodeling [42]. Experimental and epidemiological studies suggest that PFAS can promote myocardial injury and alterations in extracellular matrix composition, leading to ventricular and atrial enlargement and impaired contractility [23]. These structural changes create a substrate conducive to arrhythmias, including AF, by facilitating abnormal electrical circuits, ectopy, and heterogeneous myocardial excitability [39,41].

**Figure 3:**
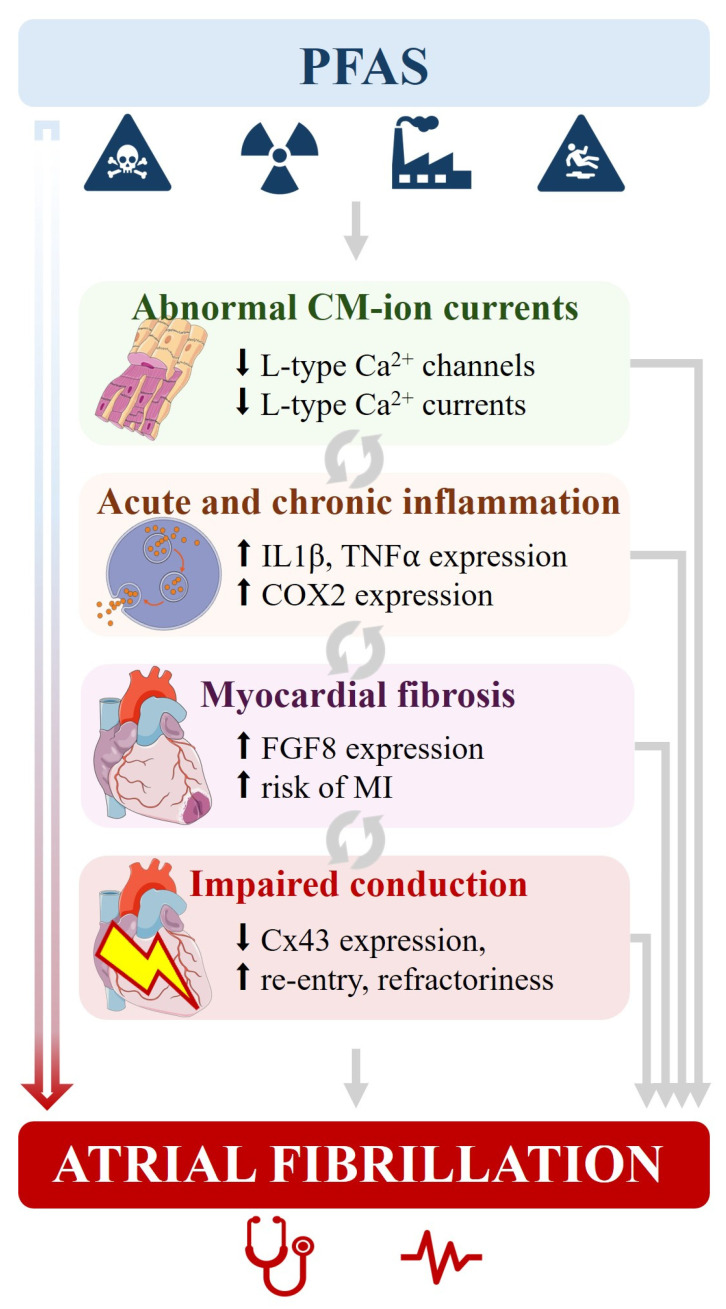
Suggested mechanisms underlying the potential risk of AF following PFAS exposure. PFAS exposure might provoke abnormal ion channel expression and activity, leading to dysfunctional cardiomyocytes (CM). As a response, CM might undergo acute and chronic inflammation which, if unresolved, can lead to cardiac fibrosis. In addition, CM injury is often accompanied by Cx43 down-expression which, coupled with myocardial fibrosis, can trigger the development of low-voltage zones and impaired conduction. Such an alteration of electrical influx generates re-entry circuits, decreases action potential duration and increases refractoriness. Altogether, PFAS-induced myocardial pathophysiology might install an arrhythmogenic substrate responsible for cardiac arrhythmias including AF. Gray arrows: established mechanisms. Red arrow: emerging effect based on preclinical or/and clinical evidence. **Abbreviations:** Ca^2+^: calcium; CM: cardiomyocyte; COX2: cyclo-oxygenase 2; Cx43: connexin 43; FGF8: fibroblast growth factor 8; IL1β: interleukin 1 beta; MI: myocardial infarction; PFAS: per- and polyfluoroalkyl Substances; TNFα: tumor necrosis factor alpha.

Cardiac structural remodeling including fibrosis, atrial dilation, and myocyte hypertrophy disrupts normal electrical conduction, while altered gap junctions and ion channel expression amplify conduction heterogeneity [21,38]. It is well-described that such changes facilitate re-entrant circuits and ectopic activity, allowing arrhythmias, including AF, to initiate and sustain [21,38,39]. Inflammatory signaling and oxidative stress often accompany structural remodeling, further destabilizing atrial electrophysiology [21,23,39] (Figure 3).

Collectively, this evidence highlights PFAS as potential environmental modulators of adverse cardiac remodeling, cardiac arrhythmias, and AF.

Overall, while PFAS-induced alterations in cardiomyocyte electrophysiology, including effects on L-type Ca^2+^ current, suggest a potential proarrhythmic profile, direct evidence of arrhythmia induction, particularly AF, remains limited, and the link to AF should therefore be considered primarily supported by indirect mechanistic pathways, warranting further in vivo and epidemiological investigation.

## 6. Comparison with Selected Non-PFAS Recalcitrant Pollutants

### 6.1. Chlorothalonil

Chlorothalonil (2,4,5,6-tetrachloro-1,3-benzenedicarbonitrile) is a broad-spectrum, non-systemic organochlorine fungicide used in agriculture and industry [43,44]. In terms of chemical structure, the benzene ring of chlorothalonil bears four chlorine atoms and two nitrile groups (-C≡N), whose electrophilic nature enables reaction with protein sulfhydryl (-SH) groups, depleting reduced glutathione (GSH) and blocking glycolysis and cellular respiration [43]. In addition, the four C-Cl bonds of chlorothalonil confer hydrolytic stability and environmental persistence [44]. Like PFAS, chlorothalonil exhibits strong adsorption to soil, primarily due to its high soil organic carbon partition coefficient (Koc) [44]. Despite low water solubility, chlorothalonil’s metabolites are more soluble and persistent, contaminating groundwater and aquatic organisms [44]. Such a hazard level led to the ban of chlorothalonil by the European Union in 2019 [45].

As an organochlorine pesticide, chlorothalonil might be associated with similar increased CVD risk and mortality than other organochlorines compounds like chlordecone, but further investigations are required to confirm such effect [46]. Furthermore, chlorothalonil was shown to inhibit Na^+^/K^+^-ATPase, an important pathophysiological pattern in CVD [47]. Similar to PFAS, chlorothalonil induces oxidative stress and mitochondrial dysfunction, contributing to cellular injury and redox imbalance [48]. Importantly, it has been suggested that chlorothalonil might act as an endocrine disruptor, with demonstrated estrogenic and thyroid-disrupting activity [48]. Altogether, these effects converge as recognized mechanisms of pesticide-induced cardiovascular dysfunction, which might reflect a potential role of chlorothalonil exposure in CVD [41] (Table 3).

Although no study has yet investigated the direct link between chlorothalonil and cardiac arrhythmias, its biohazard similarity with PFAS and other organochlorines like chlordecone suggest a potential implication in arrhythmogenesis [41,49].

**Table 3:** Selected non-PFAS persistent pollutants emerging as important risk factors for cardiovascular disease and arrhythmias.

Non-PFAS Pollutant	Chemical Formula	Source	Shared Toxicological Features with PFAS	Recognized CVD and Arrhythmogenic Substrate	Ref.
Chlorothalonil	C_8_Cl_4_N_2_	Soil, Groundwater, Aquatic organism	-Induces oxidative stress -Provokes mitochondrial imbalance	-Inhibits Na^+^/K^+^-ATPase	[47]
Desphenyl-chloridazon	C_10_H_8_ClN_3_O	Soil, Groundwater	-Provokes metabolic and cellular dysfunction -Produce persistent biologically active metabolites	-Increases systemic oxidative stress and inflammation	[50]
Chlordecone	C_10_Cl_10_O	Soil, Groundwater, Comestible organisms	-Persistent organic pollutant in soil and water -Long-term bioaccumulation in organisms -Endocrine disruption	-Alters cardiac Na^+^/K^+^-ATPase -Dysregulate cardiac Ca^2+^-ATPase -Perturbs cardiac Mg^2+^-ATPase -Increases atrial inflammation and fibrosis -Promotes atrial arrhythmias including AF	[49,51,52]
Glyphosate	C_3_H_8_NO_5_P	Soil, Water, Agricultural products	-Oxidative stress -Mitochondrial dysfunction -Potential CVD effects	-Provokes oxidative stress -Might promote inflammation and fibrosis	[53]
Dioxins	C_12_H_4_Cl_4_O_2_	Air, soil, water, food	-Chronic inflammation -Oxidative stress -Endothelial dysfunction and CVD	-Increases oxidative stress -Promotes inflammation and metabolic disturbance -Might alter Ca^2+^ handling -May provoke atrial remodeling	[54]

**Abbreviation:** AF: atrial fibrillation; ATPase: adenosine triphosphatase; CVD: cardiovascular disease; K^+^: potassium; Mg^2+^: magnesium; Na^+^: sodium; PFAS: per- and polyfluoroalkyl substances.

### 6.2. Desphenyl-Chloridazon

Desphenyl-chloridazon (DPC) is the main metabolite of chloridazon, a pyridazinone herbicide widely used in agriculture, particularly for sugar beet cultivation [55]. Due to its high polarity and strong mobility, DPC is frequently detected in both groundwater and surface water across Europe, often at higher levels than chloridazon itself [55]. DPC’s presence in groundwater often results from transfer following agricultural application [55]. Long-term lysimetric data confirm DPC persistence in soils, contributing—like PFAS—to sustained groundwater contamination [56].

Among the pathophysiological and toxicological features shared with PFAS, oxidative stress and inflammatory responses induced by DPC pesticide, as described in previous studies, suggest a potential role in increasing cardiovascular risk [50] (Table 3). To date, no experimental or clinical data has demonstrated a direct effect of DPC on cardiac conduction, myocardial ion channels, or arrhythmias including AF.

### 6.3. Chlordecone

Chlordecone (CLD), a polycyclic organochlorine pesticide, has a high lipophilicity, elevated chemical stability, and prolonged environmental persistence [49,57]. CLD intensive use in Central and South America, including banana plantations in the French West Indies between 1973 and 1993, led to long-term contamination of soil and water [57]. Similarly to PFAS, CLD bioaccumulation along food chains exposes populations mainly through diet, with high levels of detection in blood in cases of chronic exposure [57].

In terms of CVD risk, CLD was shown to alter cardiac Na^+^/K^+^-, Ca^2+^-, and Mg^2+^-ATPase activity, disrupting ionic homeostasis and perturb electromechanical stability of CM [51,52]. Prolonged exposure to CLD was shown to be associated with increased oxidative stress, inflammatory activation, and fibrotic myocardial remodeling [41,49].

In terms of cardiac arrhythmias, in vivo data revealed that prolonged exposure to CLD might provoke slowed atrial electrical conduction and increased vulnerability to arrhythmias such as AF [49]. This phenotype is accompanied by increased atrial fibrosis and activation of atrial inflammatory pathways, which characterizes a deleterious arrhythmogenic substrate [41,49]. Furthermore, in human cardiac tissue, CLD was shown to induce mitochondrial dysfunction characterized by increased oxidative stress and impaired intracellular Ca^2+^ management, two major determinants of myocardial electrical instability [58]. Taken together, these mechanisms suggest that CLD might play a role in atrial remodeling and increases the risk of arrhythmias including AF in the context of chronic environmental exposure (Table 3).

### 6.4. Glyphosate

Glyphosate, known as N-(phosphonomethyl) glycine, is an herbicide belonging to the organophosphorus family, with the formula C_3_H_8_NO_5_P [59]. Glyphosate has been widely used since 1974 as a non-selective herbicide, which acts by blocking the enzyme 5-enolpyruvylshikimate-3-phosphate synthase (EPSPS), which is essential to the vital shikimate pathway in plants and microorganisms [59]. Such inhibition prevents the production of aromatic amino acids and leads to the death of plants [59]. Comparably with PFAS, due to extensive use, glyphosate and its main metabolite, aminomethylphosphonic acid (AMPA), are found in several environmental compartments, including air, water, soil, and food [59]. In humans, glyphosate was detected and measured in urine, blood, and breast milk, indicating exposure through ingestion, inhalation, or skin contact [60].

Mounting evidence suggests that similar to PFAS, glyphosate might have harmful cardiovascular effects by provoking oxidative stress, endothelial dysfunction, impairing carbohydrate metabolism, and disruption of insulin signaling pathways [53]. Such effects are known to potentially alter cardiac function [53]. Glyphosate is also suspected of inducing myocardial remodeling promoting inflammation and fibrosis, and potential disturbance of Ca^2+^ homeostasis, which are key events altering electrical conduction and thus promoting the onset of arrhythmias, including AF [41,61] (Table 3).

### 6.5. Dioxins

Dioxins are a group of persistent chlorinated organic compounds [62]. They include polychlorinated dibenzo-p-dioxins (PCDDs), polychlorinated dibenzofurans (PCDFs), and certain dioxin-like polychlorinated biphenyl (PCBs) [54]. Among PCDDs, the most toxic and most studied compound is 2,3,7,8-tetrachlorodibenzo-p-dioxin (TCDD) [62,63]. The chemical structure of dioxins provides a high chemical stability, strong lipophilicity, and a significant capacity for bioaccumulation in tissues [64]. Dioxins are mainly produced during combustion, waste incineration, and certain industrial activities [64]. Consistent with PFAS exposure, dioxins are found in air, water, soil, and food [64]. Human exposure mainly occurs through food ingestion, but also through inhalation [63].

Dioxins act by activating the aryl hydrocarbon receptor (AhR), which alters gene expression and cell signaling pathways [54]. At the cardiovascular level, studies have shown that dioxins are associated with the development of hypertension, atherosclerosis, cardiomyopathy, and increased risk of cardiovascular mortality [54].

Among shared features with PFAS, dioxins have been demonstrated as triggers of oxidative stress, chronic inflammation, metabolic disturbance, and mitochondrial disfunction, contributing to systemic toxicity [54,63]. It has been suggested that dioxins might disrupt the electrical activity of the heart by altering Ca^2+^ handling and prolonging action potential, which may promote cardiac arrhythmias and increase the risk of AF [63] (Table 3).

## 7. Limitations

The current body of evidence remains limited by important gaps in the available data.

Longitudinal studies assessing arrhythmia outcomes, particularly AF diagnosed through continuous or long-term rhythm monitoring, are scarce in populations exposed to PFAS.

Additionally, the chemical heterogeneity and diversity of PFAS compounds complicate the attribution of specific biological effects to individual substances, while most studies focus on a limited number of well-characterized compounds.

An important limitation resides in the fact that many epidemiological investigations rely on single-time-point measurements of PFAS in serum, which may not accurately reflect long-term exposure and can introduce exposure misclassification.

Furthermore, variability in study design, exposure assessment, and outcome definitions contributes to heterogeneity across studies and limits direct comparisons.

Finally, the integration of comprehensive exposome approaches remains limited. Most studies evaluate PFAS in isolation, without accounting for combined chemical exposures or non-chemical stressors such as lifestyle, socioeconomic, or environmental factors, thereby restricting our understanding of cumulative and interactive effects on arrhythmogenesis.

Together, these limitations highlight the need for standardized methodologies, longitudinal and mechanistically integrated studies, and more comprehensive exposure assessment strategies to better define the role of PFAS in cardiac arrhythmias and AF.

Overall, this review was conducted using a narrative approach, which allows for a flexible and integrative synthesis of heterogeneous evidence spanning epidemiological, experimental, and mechanistic studies. This approach is particularly well suited to emerging fields such as PFAS-related cardiac electrophysiology, where data remain limited and diverse in nature. However, future systematic reviews and meta-analyses will be essential to quantitatively consolidate the evidence, minimize bias, and further strengthen causal inference in this rapidly evolving field associating environmental exposome including PFAS, and cardiac arrhythmias such as AF.

## 8. Conclusions

Current evidence supports the idea that prolonged PFAS exposure may represent important environmental contributors to cardiac arrhythmogenesis. Epidemiological studies, although still limited and sometimes heterogeneous, suggest associations between PFAS exposure and adverse cardiovascular outcomes, including alterations in electrocardiographic parameters. These observations are reinforced by preclinical and experimental studies demonstrating that PFAS can directly affect cardiac electrophysiology through modulation of ion channel activity, disruption of calcium handling, and induction of oxidative stress and inflammatory signaling pathways.

Importantly, mechanistic data further indicate that PFAS exposure may promote structural remodeling of the myocardium, including fibrosis, thereby creating a substrate conducive to electrical instability and arrhythmia development. The convergence of epidemiological, experimental, and mechanistic evidence thus provides a coherent framework supporting the biological plausibility that chronic PFAS exposure contributes to the initiation and maintenance of cardiac arrhythmias, including AF.

However, significant knowledge gaps remain, particularly regarding dose–response relationships, long-term exposure effects, and the direct clinical association with AF in human populations. Future studies integrating high-resolution electrophysiological approaches, longitudinal epidemiological data, and precise exposure assessment are needed to clarify these relationships.

Overall, this review highlights PFAS as emerging environmental determinants of cardiac arrhythmias and underscores the need to incorporate environmental exposure into the broader conceptual framework of arrhythmogenic disease. Addressing these challenges may improve risk stratification, inform preventive strategies, and guide public health policies aimed at reducing the cardiovascular burden associated with persistent environmental pollutants.

## 9. Take-Home Message

- PFAS are persistent, resulting in chronic human exposure with potential cardiovascular effects.
- Direct evidence for cardiac arrhythmias is limited, but associations with arrhythmogenic substrate and ECG changes are emerging.
- Experimental studies show electrophysiological disruption, including altered Ca^2+^ currents.
- PFAS may promote arrhythmogenic remodeling through myocardial inflammation and fibrosis.
- Key gaps remain, especially the lack of longitudinal studies linking PFAS exposure to clinically confirmed arrhythmias.

## Figures and Tables

**Table 1:** Clinical studies reporting PFAS exposure associated with recognized risk factors for cardiac arrhythmogenesis.

Studied PFAS	Type of Clinical Trial	Total of Participants	Recognized Risk Factor for Arrhythmia or Reported Signs of Arrhythmogenic Substrate	Ref.
Mixture of 13 PFAS	Prospective study	1229 patients	-Perturbed cardiac electrical conduction -Reduced QRS duration -Prolonged PR interval	[12]
PFOS, PFHxS	Retrospective study	46,553 patients	-Myocardial Infarction, -Advanced age (+70 years old) -Increased CVD morbidity	[13]
PFOS, PFOA	Prospective study	710 patients	-Acute coronary syndrome	[14]
PFOS	Retrospective study	101 patients	-Coronary heart disease	[13]
PFNA PFDA PFUnDA	Retrospective study	432 patients	-Congenital heart disease	[16]
PFOS PFNA	Retrospective study	7904 patients	-Elevated risk of CVD -Increased levels of circulating CRP -Augmentation of the risk of stroke	[17]
PFNA	Cross-sectional study	801 patients	-Advanced age (+70 years old) -Reduced myocardial wall thickness -Significant LV dilation -Deleterious reduction in LV mass	[18]
PFOS, PFOA	Retrospective study	42,742 patients	-Ischemic heart disease -Identified 105 genes provoking cardiovascular diseases -Genes expressed in pluripotent stem cell-derived cardiomyocyte (Casp3, Pdk4, Gdf15, Rpl17, and Ctnnb1) displayed high binding affinity with PFAS	[19]

**Abbreviation**: Casp3: caspase 3; Ctnnb1: catenin beta 1; CRP: c-reactive protein; CVD: cardiovascular diseases; Gdf15: growth differentiation factor 15; LV: left ventricle; Pdk4: pyruvate dehydrogenase kinase 4; PFAS: per- and polyfluoroalkyl substance; PFDA: perfluorodecanoic acid; PFHxS: perfluorohexanesulfonic acid; PFNA: perfluorononanoic acid; PFOA: perfluorooctanoic acid; PFOS: perfluorooctanesulfonic acid; PFUnDA: perfluoroundecanoic acid; Rpl17: ribosomal protein L17.

**Table 2:** Experimental evidence of arrhythmogenic substrate development following PFAS exposure.

Studied PFAS	PFAS’ Doses	Experimental Model	Studied Species	Recognized Arrhythmogenic Substrate Provoked by PFAS Exposure	Ref.
PFDA, PFOS, PFHxS	20 µM–200 µM	hiPSC-CMs	Human	-Altered CM mitochondrial membrane potential -Increased oxidative stress -Reduced ATP content -Increased secretion of fibrosis biomarkers -Reduced α-actinin	[23]
PFOS, PFOA, PFNA, PFHxS	1 nM–200 µM	hiPSC-CMs	Human	-Perturbed expression of genes involved in myocardial development and function	[24]
56 PFAS mixture	100 nM–100 µM	hiPSC-CMs	Human	-Prolonged QT	[25]
PFOS PFOA	1 µM–100 µM	Ventricular CM Whole-cell patch-clamp	Guinea pig	-Decreased cellular APD, increased voltage -Activated L-type Ca^2+^ current peak -Early hyperpolarization	[26]
PFOS	40 µM	ESC-CMs	Mouse	-CM mitochondrial structure damages -CM abnormal Ca^2+^ shuttle	[27]
PFOS	600 nM–20 µM	Cardiac embryogenesis	Zebrafish	-Decreased heart rate	[28]
PFOS	3.4 µM–40 µM	Cardiac embryogenesis	Oryzias melastigma	-Altered heart rate -Increased inflammatory biomarkers COX-2 and FGF8	[29]
PFOS	1 mg/kg and 10 mg/kg	Daily exposure by intraperitoneal injections	Rat	-Increased myocardial damage -Deleterious myocardial hypertrophy -Enhanced cardiac fibrosis -Cardiac inflammation (IL1β, TNFα)	[30]
PFOS	1, 5, 10, 15, 20 mg/kg (mice) 1, 2, 3, 5, 10 mg/kg (rats)	Maternal and prenatal exposure by daily gavage	Mouse Rat	-Pathological RA enlargement	[31]

**Abbreviation**: APD: action potential duration; Ca^2+^: calcium; CM: cardiomyocyte; COX-2: cyclo-oxygenase 2; ESC-CMs: embryonic stem cell-derived cardiomyocytes; FGF8: fibroblast growth factor 8; hiPSC-CMs: human induced pluripotent stem cell-derived cardiomyocytes; PFAS: per- and polyfluoroalkyl substances; PFDA: perfluorodecanoic acid; PFHxS: perfluorohexanesulfonic acid; PFNA: perfluorononanoic acid; PFOA: perfluorooctanoic acid; PFOS: perfluorooctanesulfonic acid; TNFα: tumor necrosis factor alpha.

## Data Availability

There are no new data generated or associated with this article.

## Abbreviations

The following abbreviations are used in this manuscript:

**ACS**	Acute Coronary Syndrome
**AF**	Atrial Fibrillation
**AMPA**	Aminomethylphosphonic acid
**APD**	Coronary Heart Disease
**ATP**	Adenosine triphosphatase
**Ca^2+^**	Calcium
**Casp3**	Caspase 3
**CHD**	Congenital heart disease
**CLD**	Chlordecone
**CM**	Cardiomyocyte
**COX-2**	Cyclooxygenase-2
**CRP**	C-Reactive Protein
**Ctnnb1**	Catenin beta 1
**CVD**	Cardiovascular Disease
**Cx43**	Connexin 43
**DPC**	Desphenyl-chloridazon
**ECG**	Electrocardiogram
**EPSPS**	Enzyme 5-enolpyruvylshikimate-3-phosphate synthase
**ESC-CMs**	Embryonic stem cell-derived cardiomyocytes
**FGF8**	Fibroblast growth factor 8
**Gdf15**	Growth differentiation factor 15
**hiPSC-CMs**	Human induced pluripotent stem cell-derived cardiomyocytes
**IL1β**	Interleukin 1 beta
**K^+^**	Potassium
**LV**	Left ventricle
**Mg^2+^**	Magnesium
**MI**	Myocardial Infarction
**Na^+^**	Sodium
**NLRP3**	NOD-, LRR- and pyrin domain-containing protein 3
**PCDDs**	Polychlorinated dibenzo-p-dioxins
**PCDFs**	Polychlorinated dibenzofurans
**Pdk4**	Pyruvate dehydrogenase kinase 4
**PFAS**	Per- and polyfluoroalkyl substances
**PFDA**	Perfluorodecanoic acid
**PFHxS**	Perfluorohexanesulfonic acid;
**PFNA**	Perfluorononanoic acid
**PFOA**	Perfluorooctanoic acid
**PFOS**	Perfluorooctanesulphonic acid
**PFUnDA**	Perfluoroundecanoic acid
**RA**	Right atrium
**Rpl17**	Ribosomal protein L17
**SERCA**	Sarcoendoplasmic reticulum calcium ATPase
**TCDD**	2,3,7,8-tetrachlorodibenzo-p-dioxin
**TFA**	Trifluoroacetic acid
**TNFα**	Tumor necrosis factor alpha
